# Clinical and Structural Factors Affecting Ablation Outcomes in Atrial Fibrillation Patients - A Review

**DOI:** 10.2174/1573403X19666230331103153

**Published:** 2023-07-17

**Authors:** Justin Brilliant, Ritu Yadav, Tauseef Akhtar, Hugh Calkins, Natalia Trayanova, David Spragg

**Affiliations:** 1 Division of Cardiology, Johns Hopkins Hospital, Baltimore, MD 21287, United States

**Keywords:** Atrial fibrillation, catheter ablation, arrhythmia, pulmonary vein isolation, fibrosis, thrombogenesis, atrial myopathy

## Abstract

Catheter ablation is an effective and durable treatment option for patients with atrial fibrillation (AF). Ablation outcomes vary widely, with optimal results in patients with paroxysmal AF and diminishing results in patients with persistent or long-standing persistent AF. A number of clinical factors including obesity, hypertension, diabetes, obstructive sleep apnea, and alcohol use contribute to AF recurrence following ablation, likely through modulation of the atrial electro-anatomic substrate. In this article, we review the clinical risk factors and the electro-anatomic features that contribute to AF recurrence in patients undergoing ablation for AF.

## 1 INTRODUCTION

Atrial fibrillation (AF) is the most common arrhythmia worldwide associated with significant mortality, morbidity, and poor quality of life [1]. Catheter ablation has emerged as a standard procedure for symptomatic patients with paroxysmal or persistent AF (PAF or persAF) who fail to respond to antiarrhythmic medications [2-4]. Ablation for AF has known benefits including reduced AF symptom burden and improvement in quality of life, neurocognitive function, mood, and functional status [5-7]. In patients with impaired LV systolic function, ablative therapy for AF has been shown to reduce heart failure hospitalization, improve LV ejection fraction, and reduce overall mortality compared to patients treated medically [2, 4, 8-11]. While the benefits of AF ablation are clear, recurrence rates of AF following ablation are problematic. In patients with PAF, complete elimination of recurrent arrhythmia after pulmonary vein isolation (PVI) occurs in just over 50% of patients, though the reduction of AF burden is more pronounced (typically 99%) [8]. In patients with persistent or long-standing persistent AF, recurrence rates are even higher (as great as 60%) [12, 13]. Identifying modifiable clinical risk factors that impact AF recurrence is critical to optimizing success in patients undergoing invasive management of AF. Here, we provide a brief discussion of the pathophysiology of AF, a review of the electro-anatomical remodeling that occurs with AF, and finally, a look at the clinical risk factors that contribute to AF susceptibility with an emphasis on how those risk factors may contribute to adverse electro-anatomical remodeling. Our aim is to underscore the criticalimportance of risk factor modification in patients considering rhythm-control therapy for AF.

## 2 PATHOPHYSIOLOGY OF AF

AF is a trigger-and-substrate arrhythmia. Rapid, repetitive firing from ectopic atrial foci, most typically located in the ostia of the pulmonary veins (PVs), induces paroxysms of AF. Atrial tissue in patients with PAF is relatively healthy, allowing for normal conduction velocities and refractory periods and resulting in relatively brief episodes of AF. Anatomical changes in the atria, including interstitial fibrosis and chamber enlargement, contribute to deranged electrical propagation through the atrium and the shortening of atrial-effective refractory periods (AERPs). These electro-anatomical changes allow for episodes of AF that are more easily induced and more durable. As AF progresses from a principally trigger-driven to a more substrate-maintained arrhythmia, patients evolve from experiencing PAF to experiencing persistent or (eventually) long-standing persistent AF [14-16]. This change in fundamental pathophysiology explains in part why catheter ablation for AF (typically in the form of trigger elimination by PVI) is relatively successful in PAF patients (in whom triggers play a critical role) but less so in patients with persAF (in whom lasting AF may be induced by less aggressive ectopic sites than those found in the PV ostia).

Atrial structural and electrical remodeling plays a central role in the progression of paroxysmal AF to persistent or long-standing persistent AF by allowing for the maintenance of *functional reentry* in the atrium. Depolarizing wavefronts move chaotically through fibrillating atrial tissue and require new, repolarized tissue to find and excite. Rapidly conducting wavefronts and persistently refractory tissue result in a head-meets-tail phenomenon where no repolarized atrial tissue can be found, which results in the termination of the AF episode. Reduction in conduction velocity and refractory periods conspire to allow functional reentry to persist, leading to prolonged AF episodes. In addition, regions of fibrotic tissue interspersed with normal atrial myocardium may give rise to focal reentrant drivers. These pathologic alterations allow for more persistent AF episodes [17, 18], which in turn lead to further fibrosis and augmented repolarization – a positive feedback loop captured by the phrase “AF begets AF”. Whether the restoration of normal sinus rhythm allows for complete reverse remodeling of the atrium is not clear [19, 20], but atrial substrate remodeling clearly plays a critical role in rendering treatments for AF less effective. Understanding the electro-anatomical factors contributing to AF susceptibility and how clinical risk factors drive electro-anatomical remodeling is central to an effective rhythm-control strategy for AF.

## 2 ELECTRO-ANATOMICAL REMODELING

### 2.1 Left Atrial Dilation and Fibrosis

**Fig. (1):**
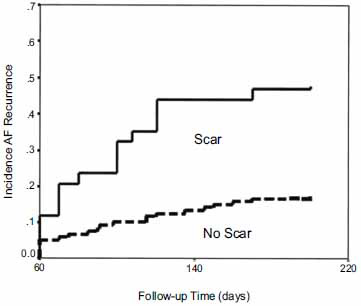
Kaplan-Meier curves depicting late atrial fibrillation (AF) recurrence in patients with and without left atrial scarring (LAS) over time. Patients with LAS have a significantly increased incidence of late AF recurrence compared with patients without LAS (p < 0.01 by log-rank).

Previous studies have shown that AF is associated with left atrial (LA) dilation. LA dilatation is a structural remodeling process in response to long-standing pressure and volume overload; it can occur commonly in diastolic dysfunction, left ventricular hypertrophy, mitral valvular disease, and hypertension [21, 22]. Chronic LA enlargement is tied pathologically to LA fibrosis, inflammation, thrombogenesis, atrial myopathy, and to potentially irreversible changes in the atrial substrate (Fig. **1**) [23]. Clinically, these irreversible changes have been linked to subsequent failure in rhythm-control approaches for AF. A recent meta-analysis correlated LA dilation to increased AF recurrence following ablation [24]. In the analysis, 3750 patients from 22 separate investigations were included in a weighted mean difference (WMD) calculation of LA diameter. Patients with arrhythmia recurrence following ablation had larger LA diameters (WMD of 1.87mm; 95% CI 1.26-2.48, p < 0.001) than those free of AF at 6 months, 12 months, and > 12 months after catheter ablation.

How LA size increase promotes AF is unknown. One explanation invokes augmented atrial fibrosis [25]. Increased LA diameter results in mechanical stretch, inducing collagen synthesis and both angiotensin II (Ang II) and transforming growth factor β (TGF-β) production in cardiac fibroblasts [26-28]. Increased expression of Ang II and TGF-β, in turn, upregulates extracellular-matrix (ECM) protein synthesis and secretion to ultimately result in progressive atrial fibrosis [29-31]. In addition, a mechanical stretch of these same fibroblasts may directly modulate myocyte electrical activity to promote electrical remodeling [32]. Circulating inflammatory markers including C-reactive peptide (CRP), interleukin-6 (IL-6), IL-1, myeloperoxidase (MPO), and tumor necrosis factor-α (TNF-α) positively correlate with the presence of AF, the progression from PAF to persAF, and with progressive LA dilation [30-34].

To clarify that LA fibrosis in the setting of AF reflects a local process, rather than a purely systemic one, Hohmann *et al.* showed in LA appendage tissue that inflammatory CD3 cells significantly increased in patients with PAF and persAF compared to those in sinus rhythm [35]. Future studies are needed to elucidate targets in the specific pathways mediating LA stretch, enlargement, and fibrosis.

### 2.2 LA Fibrosis in the Clinical Setting

**Fig. (2):**
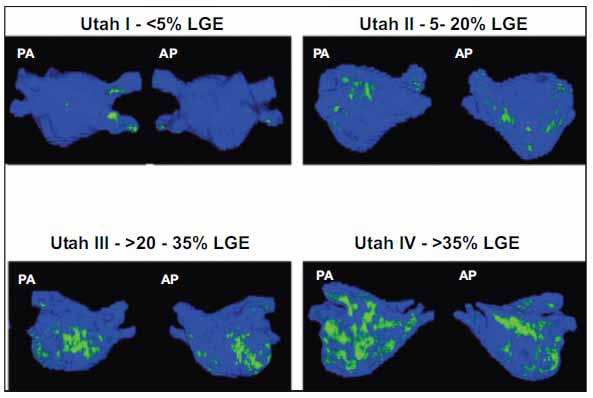
University of Utah Atrial Fibrillation LGE-MRI-based staging system. Utah I ≤ 5% atrial enhancement, Utah II >5–20%, Utah III > 20–35%, and Utah IV > 35%. The post-AF ablation recurrence rate was proportional to the amount of pre-AF ablation LA wall enhancement: there were no recurrences in stage 1, there was a 28% recurrence rate in stage 2 patients, a 35% recurrence in stage 3, and a greater than 50% recurrence in those patients with stage 4.

Atrial fibrosis is an important pathophysiological factor that contributes to the maintenance of AF. Assessment of LA scarring (LAS) has proven to be a useful means of predicting response to ablation for AF. Verma *et al.* found that pre-existent LAS is an independent predictor of procedural failure [36]. In 700 patients undergoing first-time PVI, all patients underwent detailed LA voltage mapping prior to ablation. LAS was defined as bipolar voltage amplitude ≤ 0.05 mV in multiple contiguous LA locations. Patients were followed for a mean of 9 months after ablation for arrhythmia recurrence. Of 700 patients, 42 had LAS with an average of 21+/- 11% of the LA surface involved; these patients showed significantly higher AF recurrence (57%) compared to non-LAS patients (19%) (p=0.003; Fig. **2**). LAS was also associated with significantly larger LA size, lower EF, and increased inflammatory markers. On multivariate analysis, LAS was found to be the only independent predictor of recurrence (hazard ratio 3.4, 95% confidence interval 1.3 to 9.4; p = 0.01).

**Fig. (3):**
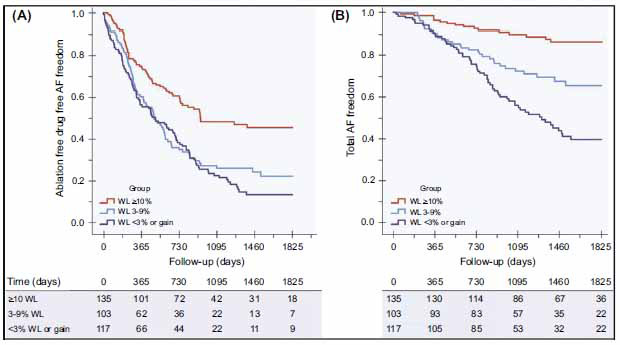
Long-term effect of goal-directed weight management in an atrial fibrillation cohort: A long-term follow-up study. Atrial fibrillation freedom outcome according to group. (**A**) Kaplan–Meier curve for atrial fibrillation-free survival without the use of rhythm control strategies. (**B**) Kaplan–Meier curve for atrial fibrillation-free survival for total atrial fibrillation-free survival (multiple ablation procedures with and without drugs). **Abbreviations:** WL, weight loss.

The determination of LA fibrosis by invasive electro-anatomical mapping (EAM) has significant drawbacks. Because it is performed during the ablation procedure itself, invasive scar mapping does not provide pre-procedural data that might guide clinical decision-making (including the determination that ablation may be futile). Furthermore, there are no uniform standards for what constitutes a scar among ablation operators [37]. Cardiac MRI (cMRI) with delayed gadolinium enhancement (DGE-cMRI) to delineate regions of LA scar has emerged as a useful tool in pre-procedure LA fibrosis assessment [38-40]. Marrouche *et al.* developed a pre-ablation scoring system based on the LAS burden seen on DGE-cMRI. Patients were assigned a “Utah score” of 1, 2, 3, or 4, correlating with LA scar percentages of ≤ 5% enhancement, >5–20%, > 20–35%, and > 35% (Fig. **3**). Patient outcomes following ablation correlated inversely with Utah score, with worse outcomes seen in those patients with more scar [41].

A subsequent study, DECAAF I, was a multicenter, prospective cohort study investigating LA fibrosis assessment using pre-procedure DGE-cMRI prior to ablation. A total of 319 patients underwent DGE-cMRI 30 days prior to ablation to estimate the LA fibrosis; 260 patients were followed after ablation. The authors found an inverse, linear correlation between the amount of atrial fibrosis and the incidence of recurrent arrhythmia following catheter ablation for patients with both PAF and persAF [39].

The DECAAF II trial, a prospective, randomized, multicenter trial testing the safety and efficacy of targeting regions of LA scar for ablation, was recently completed. In this trial, 843 patients with persistent AF undergoing index AF ablation were randomized in 1:1 to receive conventional PVI alone (422) *vs* PVI +fibrosis-guided ablation (421). All patients underwent LGE-MRI before the ablation procedure to delineate LA fibrosis. Patients were followed for 12-18 months after ablation for arrhythmia recurrence. No significant differences in atrial arrhythmia recurrence between the PVI group *vs* PVI + fibrosis-guided ablation group were seen. Additional prospective studies will need to clarify the role of targeting specific areas of fibrosis to improve outcomes following catheter ablation.

Progressive atrial fibrosis after failed ablation for AF may contribute to further entrenchment of fibrillation by continuing adverse structural remodeling and by giving rise to new fibrotic areas capable of supporting fibrillatory activity [42]. The cornerstone of ablation for both paroxysmal and persistent AF is PVI. Pulmonary vein reconnection results in frequent recurrence of AF despite ablation, however, allowing for the continuation of the same structural changes (dilation, fibrosis) characteristic of the pre-ablation period. This more advanced fibrosis may render further attempts at rhythm control less effective [43].

### 2.3 Electrical Remodeling

In both human and animal studies, AF has been linked to deranged atrial electrophysiology. The two principal electrical changes that give rise to AF have reduced conduction velocity and shortening of atrial refractoriness. Both of these changes promote ongoing functional reentry by reducing the chance of head-meets-tail extinguishment of an arrhythmic episode. How AF changes both atrial conduction velocity and refractory periods has been investigated but is not completely elucidated.

Conduction velocity in diseased atrial tissue can be reduced by scarring and interstitial fibrosis (as discussed above), but also by direct electrical uncoupling of atrial myocytes [44]. Atrial myocytes are linked electrically by gap junctions, composed of transmembrane ion-channel proteins called connexins [45]. It has been hypothesized that induction of AF leads to progressive downregulation of specific connexin proteins including connexin40 (Cx40) and connexin 43 (Cx43) resulting in attendant uncoupling of atrial myocytes, thus suggesting a plausible mechanism for the reduction in conduction velocity [46-48]. Ausma *et al.* showed that the distribution of Cx40 in goat atrial myocardial cells changed from a homogenous distribution in sinus rhythm to a heterogeneous pattern when in AF for 4 months, with areas in the right and left atria almost devoid of these protein levels [49]. After restoration of sinus rhythm for 2 months, Cx40 levels had normalized completely back to a homogenous distribution. Transgenic porcine models of adenovirus expressing Cx40 and Cx43 have shown that induced increases in these proteins were associated with improvements in interatrial conduction velocity and with a reduced propensity for persAF, suggesting a mechanistic link between connexin expression and AF [50, 51]. Furthermore, small-molecule drugs that promote gap junction conductance show improvement in conduction velocity in some dog models and may be actionable targets for AF treatment in the near future [52, 53].

AF leads to changes not only in atrial conduction but also in myocyte repolarization. Atrial myocyte repolarization is driven by the duration of the plateau phase during the action potential and is mediated largely by the calcium current in L-type calcium channels [54]. During AF, intracellular calcium accumulation inactivates these L-type calcium channels and decreases transmembrane calcium gradients, reducing the magnitude and duration of the action potential plateau [55, 56]. In a goat model of pacing-induced AF, Wijffels *et al.* showed that AF episodes as brief as 24h lead to progressive shortening of the atrial action potential by 12 to 35% [57]. Augmented repolarization was mediated by the likely downregulation of L-type calcium channels, with a 73% reduction of calcium current density compared to sinus rhythm models [58]. Martins *et al.* showed in a sheep model that the action potential duration and atrial refractory period shorten *via* the decrease in L-type calcium channels and increase in inwardly rectifying potassium channels when transitioning from paroxysmal to persistent AF [59].

Finally, there may be other electrical mechanisms (in addition to reduced conduction velocity and augmented repolarization) by which AF is perpetuated. Regions of scarring and low-voltage areas may give rise to regions of high dominant frequency firing that helps drive ongoing AF. During AF, low voltage areas appear to correlate with high dominant frequency firing, complex fractionated electrograms, and rotor activity [60]. One study found that triggers from the pulmonary vein and superior vena cava showed reduced voltage in the pulmonary vein antra and right atrium, respectively [61]. However, a majority of high dominant frequency sites were shown not to be correlated with low voltage sites in sinus rhythm and may only be measured in AF [62, 63]. Identifying a direct relationship between low voltage areas and either electrogram fractionation or rotors have been difficult and not well established in the literature [60]. However, the number of patients in prior studies with left atrial low voltage areas had only been 10% in PAF and 35% in persAF [64, 65]. The recent volcano RCT investigated the efficacy of left atrial low voltage ablation in addition to PVI on rhythm outcomes in paroxysmal AF; although the presence of left atrial low voltage areas predicted lower AF recurrence-free rate, ablation of these areas in addition to PVI had no beneficial impact on 1-year rhythm outcomes [64]. The upcoming results of the SUPPRESS AF RCT will investigate low-voltage ablation strategies in patients with persistent AF undergoing their first catheter ablation to inform future treatment strategies [66].

It is possible that restoration of sinus rhythm prior to AF ablation leads to reverse electrical remodeling and improves ablation outcomes. In particular, amiodarone has been explored in canine models of AF and has been shown to prevent action potential shortening, depressed conduction velocity, interstitial fibrosis deposition, and AF inducibility [67, 68]. Utilizing this pre-ablation conditioning strategy, Benak and colleagues investigated 62 persAF patients treated with amiodarone and cardioversion three months prior to anticipated PVI [69]. They selected the patients who maintained normal sinus rhythm on amiodarone and performed PVI, finding comparable ablation results to a matched cohort of patients with PAF at 6 months and 12 months following ablation. Furthermore, the use of dofetilide to reduce the burden of AF prior to ablation led to a decreased recurrence rate and had been associated with a decrease in P wave duration [70]. However, larger studies that incorporate pre-ablation conditioning with antiarrhythmic therapies and/or electrical cardioversion to restore normal sinus rhythm prior to ablation will need to be performed to further study the degree of atrial electrical remodeling together with outcomes following catheter ablation.

## 3 CLINICAL RISK FACTORS CONTRIBUTING TO AF SUSCEPTIBILITY

### 3.1 Obesity

**Fig. (4):**
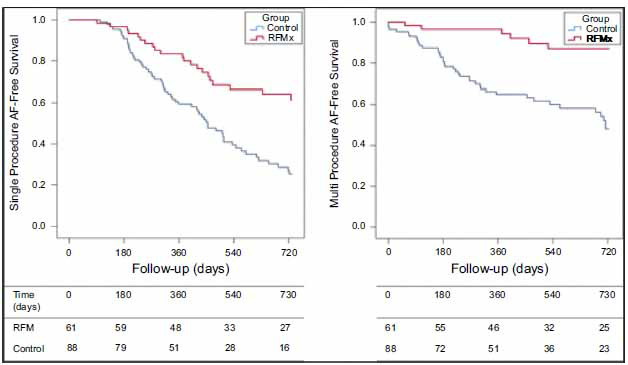
Kaplan-Meier curves for single-procedure, drug-free, AF-free survival **(left)** and for total AF-free survival (multiple procedures ± drugs) **(right)**. Curves for 2 years are provided, after which <20% of patients completed follow-up. Note that data are provided after the last procedure using a 3-month blanking period. **Abbreviation:** RFM = risk factor management.

Several studies have established that overweight and obese populations have higher incidence, prevalence, severity, and progression of AF as compared with their normal-weight counterparts [71, 72], raising the possibility that obesity may be a modifiable risk factor that could impact AF incidence and recurrence. The LEGACY trial investigated the long-term impact of weight loss on AF symptoms and burden [73]. A total of 355 patients were offered goal-directed weight management strategies and followed for a mean of 4 years. Based upon the proportion of total body weight loss, patients were divided into 3 groups (Group 1: >10% of total body weight lost; Group 2: 3-9%; Group 3: <3%). Weight fluctuation between visits was evaluated as well, with patients categorized as having linear weight loss, weight fluctuation, or no weight loss. Patients underwent catheter ablation and/or received antiarrhythmic drugs for rhythm control. On follow-up, 45.5% of Group 1, 22.2% of Group 2 and 13.4% of Group 3 (p< 0.001) patients remained arrhythmia-free off any antiarrhythmic drugs or ablation; furthermore, total arrhythmia-free survival rates were 86.2% in group 1 compared with 65.5% in group 2 and 39.6% in group 3 (p<.001) (Fig. **4**). On multivariate analysis, >5% weight fluctuation was associated with a significantly higher risk of AF recurrence as compared to <2% weight fluctuation (p=0.02, C.I- 1.0-4.3,95% confidence interval). In addition, stable weight loss was associated with improvement in structural remodeling including a reduction in left atrial volume and left ventricular hypertrophy, reduction in inflammatory biomarkers, and improvement in overall symptom scores.

**Fig. (5):**
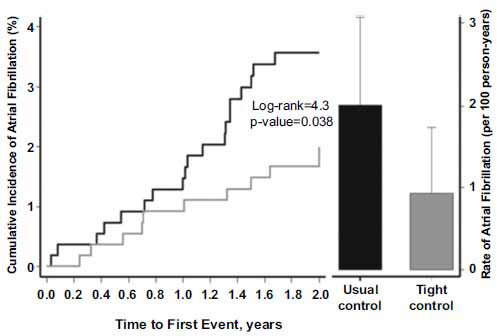
Incidence of new-onset atrial fibrillation in patients in hypertensive patients randomized to a a target systolic blood pressure goal of < 140 mm Hg (usual control) or < 130 mm Hg (tight control) in the Cardio-Sis randomized controlled trial.

A second cohort study – ARREST -AF (Aggressive risk factor management for Atrial fibrillation and implication for the outcome of ablation) – investigated the impact of risk factor management specifically on AF ablation procedure outcomes [74]. Out of total 149 patients with BMI > 27 kg/m2 and > 1 cardiac risk factor undergoing catheter ablation, 61 patients underwent risk factor management and 88 did not (control group). Risk factor management (RFM) included a goal-directed weight loss program along with a moderate-intensity exercise regimen, strict blood pressure control with SBP < 130 mm Hg, lipid control with LDL < 100, glycemic control with HbA1c < 6.5%, alcohol reduction to < 3 standard drinks/week, and treatment of OSA with positive airway pressure. Patients with RFM had greater freedom from AF recurrence as compared to the control group, with initial (32.9% *vs* 9.7%) and repeat (87% *vs* 17.8%) AF ablation with median follow-up 42.8 for the RFM group and 42.4 months for the control group (Fig. **5**). While clinical characteristics were grouped and optimized together, confounding analysis of the relative contributions of each risk factor, and multivariate analysis suggested that type of AF (p<0.001) and RFM (p<0.001) were independent predictors of arrhythmia-free survival.

Specific mechanisms linking obesity to AF propensity include not only structural remodeling of left atrial diameter and left ventricular hypertrophy (both correlate with obesity), but also epicardial fat volume. Epicardial fat is an independent predictor of incident AF risk [75]. Left atrial epicardial fat volume is associated with a left atrial size and may serve as metabolically active tissue rich in adipokines, pro-inflammatory cytokines, and growth factors that alter neighboring myocardium [76, 77]. Epicardial fat may perpetuate AF through direct inflammatory effects on adjacent LA tissue, as suggested by the close correlation between epicardial adipose tissue and high dominant-frequency sites during AF, suggesting a focal rotor or local reentry site [78]. In addition, epicardial fat has been associated with low-voltage atrial zones, possibly through fatty deposits in the myocardium, leading to conduction abnormalities and a corresponding predisposition for AF [79]. Therefore, obesity and targeted interventions for weight loss may reduce the AF burden by reducing epicardial adipose tissue and the effects of that tissue on atrial electrophysiology.

### 3.2 Hypertension

**Fig. (6):**
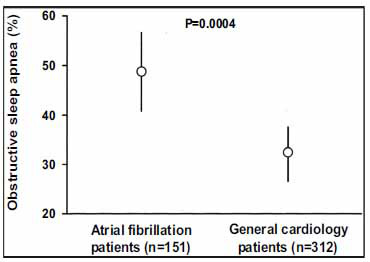
Proportion and 95% CI of patients with OSA. Prevalence of OSA is significantly higher in patients with AF than in patients without past or current AF in general cardiology practice (49% [95% CI 41% to 57%] *vs* 32% [95% CI 27% to 37%], *p*=0.0004).

In addition to obesity, hypertension has been known to be an independent risk factor for the development of AF in various epidemiological studies and has been associated with left atrial diameter and left ventricular wall thickness [80-83]. In the ARIC prospective study, elevated systolic blood pressure ≥ 140 mm Hg or diastolic blood pressure ≥ 90 mm Hg was the most important risk factor driving AF burden over a mean follow-up of 17.1 years, explaining 20% of all AF incidence [84]. In addition to the new onset of AF, hypertension was an independent predictor of AF progression from paroxysmal to persistent or permanent AF after one year of follow-up [85]. Intensive control of blood pressure to decrease the incidence of AF has been investigated. In the cardio-Sis randomized controlled trial [86], patients with hypertension were randomly assigned to a target systolic blood pressure goal of < 140 mm Hg (usual control) or < 130 mm Hg (tight control); the onset of new AF was a secondary outcome of the study. At a median follow-up of 2 years, the onset of new AF occurred in 3.8% of patients in the usual control group compared to 1.8% of patients in the tight control group (p = .044) [86] (Fig. **6**). In the SPRINT randomized controlled trial of intensive systolic blood pressure control (< 120 mm Hg) *versus* routine blood pressure control (< 140 mm Hg), a secondary analysis showed a 26% lower risk of developing new AF with intensive control (hazard ratio, 0.74 [5%CI, 0.56-0.^98^]; p=0.037) during a follow up of 5.2 years [87]. Early diagnosis and treatment of hypertension appear to have significant therapeutic implications for a reduction in AF burden.

The pathophysiology in patients with hypertension that underlies the progression from sinus rhythm to AF can be explained by the combination of structural and electrical remodeling. In several experimental studies that induced hypertension in rats and sheep, structural changes such as left atrial hypertrophy, inflammation, and fibrosis were seen [88, 89]. Furthermore, electrical remodeling including conduction abnormalities such as shortening of left atrial wavelength, slower conduction velocity, reduced expression of connexin 43 (found in gap junctions), and increased inducibility and duration of AF have been demonstrated as a consequence of experimentally induced hypertension [88, 90-92]. In humans, atrial remodeling in hypertension from left ventricular hypertrophy has been characterized by global conduction slowing, increased areas of low voltage, and increased AF inducibility [93].

The renin-angiotensin-aldosterone has been shown to have pro-inflammatory actions resulting in atrial myocyte hypertrophy and electrophysiologic changes that may perpetuate AF [91, 94]. In a prior meta-analysis, the use of angiotensin-converting enzyme inhibitors (ACE-Is) and angiotensin receptor blockers (ARBs) reduced the relative risk of AF by 28% (p=.00002) with significant reductions seen in heart failure and left ventricular hypertrophy [95]. However, modulation of this pathway has not been shown to definitively improve catheter ablation outcomes in paroxysmal or persistent AF [96]. Patients with paroxysmal or persistent AF in the SMAC-AF trial, for instance, were divided into standard blood pressure control (< 140/90 mm Hg) *versus* aggressive blood pressure control (< 120/80 mm Hg) between 0 and 6 months before catheter ablation and 3 months following catheter ablation, with the majority of patients treated with ACE-Is and ARB [97]. The primary outcome of symptomatic recurrence of AF, atrial tachycardia, or atrial flutter occurred in 61% of patients in both groups (hazard ratio, 0.58 [95%CI, 0.34-0.^97^]; p=0.763) during a median follow up of 14 months.

Additional studies are needed to elucidate the underlying mechanisms between the treatment of hypertension and recurrence of AF following catheter ablation to best define optimal blood pressure targets, anti-hypertensive regimens, duration of therapy, and adjunctive procedural strategies.

### 3.3 Obstructive Sleep Apnea

**Fig. (7):**
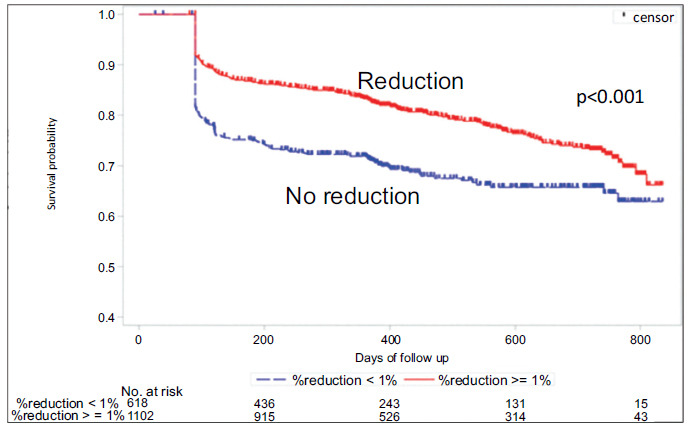
Kaplan-Meier analysis of atrial fibrillation/atrial tachycardia recurrence-free survival. *p* values were determined by the generalized Wilcoxon test. Red and blue lines indicate patients who reduced alcohol consumption by ≥1% and <1%, respectively.

The links between obstructive sleep apnea (OSA) and AF, like those between obesity and AF, have been established through a number of observational investigations [98-101]. OSA is found more often in patients presenting for AF therapy than in general cardiac patients without documented AF [98]. A prospective study was performed on the patients undergoing electrical cardioversion for AF (n=151), and consecutive patients without a history of AF were referred to a general cardiology group (n=321), all of whom were screened for OSA. Patients in each group had similar underlying clinical characteristics and comorbidities, but the proportion of patients with OSA in the AF group was significantly higher than that in the general cardiology group (49% *vs* 32%; p=0.0004) [98-101] (Fig. **7**).

Similar findings were reported in a larger observational cohort of 3,450 patients undergoing OSA evaluation *via* polysomnography and cardiac care [102]. Patients without an AF diagnosis were followed for 5 years following initial screening for OSA. Incident AF in newly diagnosed OSA patients was significantly higher than in those without OSA (4.3% *vs* 2.1%, p = .002) during 4.7y of follow-up. A number of clinical risk factors (age, male gender, CAD, and BMI) were independently associated with AF risk, including OSA severity (even after correcting for obesity) [102].

Not only is OSA frequently diagnosed in AF patients, but untreated OSA has also been shown to negatively impact ablation outcomes in patients undergoing PVI. In a meta-analysis of AF ablation patients with OSA, the risk of AF recurrence after catheter ablation is 25% greater in OSA patients as compared to non-OSA controls (RR 1.25,95% CI 1.08 to 1.45, p=0.003) [103]. In another meta-analysis, the use of CPAP in AF ablation patients with known OSA was associated with a significant reduction in AF recurrence following ablation (by 42%) compared to OSA patients who did not use CPAP (relative risk: 0.58; 33.3% *vs* 57.6%) [104]. These findings suggest that interventions for OSA management may improve ablation outcomes and support broad screening for OSA in AF ablation patients. However, OSA is significantly underdiagnosed and untreated in AF despite its high prevalence. Therefore, the 2017 AF Consensus Statement recommends broad screening for OSA in patients considering catheter ablation for AF2. OSA evaluation should be performed with a portable sleep study rather than by history or screening questionnaires to improve accurate diagnosis [105].

The mechanisms by which OSA may contribute to AF have been investigated. OSA has been shown to modify the atrial substrate in ways that predispose it to AF. Dmitri and colleagues performed basic EP studies and catheter ablation in 40 AF patients with OSA and 20 AF patients without; both groups had no differences in the prevalence of other AF risk factors [106]. Compared to non-OSA AF patients, the OSA cohort was found to have prolonged conduction time along the coronary sinus and in the right atrial tissue (p=0.9), longer P wave duration (p=0.01), longer corrected sinus node recovery time (p=0.02), lower atrial voltage in both atria (p=0.001), slower conduction velocity (RA, P=0.001; LA, p=0.02), and more wide-spread complex electrograms in both atria (RA, p= 0.02; LA, p=0.01). This suggests that OSA in AF patients is associated with significant atrial enlargement, conduction abnormalities, reduction in voltage, and longer sinus node recovery.

OSA may also drive increased triggering activity acutely during apnea episodes, additionally increasing AF propensity. Linz and colleagues studied the relationship between acute obstructive respiratory events (OREs) and the occurrence of premature atrial contractions (PACs) in patients with recurrence of AF following cardioversion [107]. They found that PAC burden was significantly increased during OREs (7±2 per 10 seconds) in 40 patients during deep sedation within 2 minutes after cardioversion with the restoration of sinus rhythm. After 2 minutes, nasopharyngeal tube insertion to prevent OREs led to a clear reduction in PAC burden (2 ± 0.5 per 10 seconds; p < 0.0001). PACs were low (1±1 per 10 seconds) in the 32 patients who did not exhibit OREs. There was a significant difference noted in AF recurrence within the first week of cardioversion between patients with and without OREs (p=.016), with notable shortening of the mean coupling intervals of PACs among patients with OREs who relapsed (p=.0013). Furthermore, in a pig model after atrial stimulation to replicate electrical remodeling, maneuvers meant to replicate OSA *via* a negative tracheal pressure device led to an immediate, transient reduction in AERP (117±12 ms *vs* 75±9 ms, p=.001) and increased the number of PACs by 230% in the first 2 minutes after termination of AF [107]. The acute changes in intra-thoracic pressure, hypoxemia, and sympathovagal activation during apnea episodes lead to a combination of atrial triggers and shortening of the AERP to facilitate a plausible electrophysiologic mechanism for the maintenance of AF [108, 109].

### 3.4 Alcohol

The relationship between alcohol consumption and AF is so well established that it has its own moniker – “holiday heart.” However, the mechanisms by which alcohol contributes to the pathogenesis of AF are not completely understood but likely are mediated by effects of both trigger activity and atrial conduction properties that help drive fibrillation [110].

Acute alcohol intoxication can cause specific electrophysiologic effects that may facilitate AF induction and maintenance. In various animal models, acute intoxication of alcohol resulted in the shortening of atrial action potential duration through the upregulation of specific potassium channels leading to repolarization and demonstrating increased AF susceptibility after burst atrial pacing [111-113]. In a murine model, Zhang *et al.* showed, in both acute and chronic alcohol intoxication, a dose-related response of increased AF vulnerability with decreased conduction velocities in both atria and shortened AERPs (with increasing dispersion) only in the right atria [114]. In addition, alcohol has been hypothesized to trigger and induce AF *via* changes in the discharge of both the sympathetic and parasympathetic nervous systems, but isoproterenol administration during alcohol infusion did not lead to more AF [115, 116].

In addition to electrical remodeling, habitual consumption of alcohol may result in AF progression because of myocardial injury and structural remodeling. The mechanisms by which alcohol and its metabolite acetaldehyde act as cardiac toxins include oxidative stress, mitochondrial dysfunction, apoptosis, and abnormal fatty acid metabolism [117, 118]. Alcohol may also impair excitation-contraction coupling through the inhibition of calcium release from the sarcoplasmic reticulum and reduce myofilament calcium sensitivity and an attenuated response to inotropes [119, 120]. These molecular mechanisms may explain how chronic alcohol consumption leads to structural remodeling.

Reduction in alcohol intake has been shown to reduce the AF burden. In a recent RCT among 140 AF patients who consumed 10 or more drinks per week, 70 patients reduced their alcohol intake by 87.5% (designated ‘abstinent group’), and the other 70 patients reduced their alcohol intake by 19.5% (designated ‘control group’) [121]. After a 2-week blanking period, AF recurred in 53% of abstinent patients *vs* 73% in the control patients after 6 months (HR, 0.55; 95% CI, 0.36-0.84, *p*=.005). During this 6 month's follow-up, AF burden was significantly lower in the abstinent group (median 0.5%, [interquartile range (IQR), 0.0 – 3.0] *vs* control (median 1.2%, [IQR, 0.0-10.^3^], *p*=.01).

The role of alcohol consumption in patients undergoing AF ablation has also been explored. In an observational study, a total of 122 patients with paroxysmal AF who underwent PVI and LA voltage mapping during sinus rhythm were classified as alcohol abstainers, moderate drinkers, or heavy drinkers based on their daily alcohol consumption [122]. Patients were followed up for 20.9±5.9 months for arrhythmia recurrence. Forty patients (35.1%) experienced AF recurrence. The success rate was 81.3%, 69.2%, and 35.1% in alcohol abstainers, moderate drinkers, and heavy drinkers, respectively (overall log-rank, P<0.001). Multivariate analysis showed that both alcohol consumption and low-voltage index were independent predictors of AF recurrence (hazard ratio [HR], 1.579; 95% CI, 1.085–2.298; *p*=0.017; HR, 2.188; 95% CI, 1.582–3.026; P<0.001, respectively).

Alcohol intake as a modifiable risk factor was investigated in a recent multicenter, prospective observational study [123]. Patients undergoing catheter ablation were enrolled and were advised to limit their alcohol intake to < 20g/Week after the procedure. Patients were followed up for a mean of 1 year after ablation for any recurrence, and a percentage reduction of alcohol consumption from baseline to recurrence or to 1-year post-ablation (if no recurrence) was calculated. The authors found that alcohol reduction of ≥ 1% after ablation was associated with a 37% lower rate of AF/AT recurrence overall (hazard ratio, 0.63, p <0.001, 95 CI – 0.518- 0.768), with the greatest benefit in patients whose baseline alcohol consumption was ≥ 120 g/week, who saw a 44.5% lower rate of AF/AT recurrence.

### 3.5 Diabetes

Diabetes is a final well-known contributor to the risk for the development of AF, with diabetic patients at a 39% greater risk of developing AF compared to matched, non-diabetic patients [124]. Diabetes is an independent contributor to atrial fibrosis, likely through increased oxidative stress, growth factor expression, advanced glycation end products, and the resultant inflammatory response in cardiac tissue. In addition, hyperglycemia itself of the sort seen in diabetic patients has been shown to contribute directly to tissue fibrosis and atrial remodeling [125] In human atrial tissue, type-1 collagen expression was significantly elevated in fibroblasts in type-2 diabetes compared to those without type-2 diabetes [126]. Furthermore, hyperglycemia seen in type-1 and type-2 diabetes showed associated elevations in pro-fibrotic mediators including angiotensin II and TGF-β signaling in addition to reactive oxygen species (ROS) production; likewise, angiotensin-converting enzyme inhibitors reduce both collagen and TGF-β production in type-1 and type-2 diabetes [127-129]. These changes in turn may also lead to electrical remodeling with significantly longer atrial activation times and lower bipolar voltages as seen in patients with altered glucose metabolism during catheter ablation [130].

Catheter ablation should be offered to patients with AF and diabetes who have already been trialed on anti-arrhythmic therapy. Forleo *et al.* showed in an RCT that patients with both AF and diabetes had less recurrence of AF if offered catheter ablation *vs* the sole addition of another anti-arrhythmic drug after one year of follow up (80% *vs* 42.9%, p = 0.001) [131]. Furthermore, in regards to the success for catheter ablation in patients with AF and diabetes from a meta-analysis of 1464 patients, higher basal glycated hemoglobin level was associated with a greater incidence of arrhythmia recurrences at a mean follow-up of 27 months [132]. Therefore, treatment of diabetes has been associated with improved catheter ablation outcomes.

Several studies have shown that glucose management with oral hypoglycemic agents in patients with diabetes can reduce the propensity for AF. Whether this mechanism of action is through the reverse remodeling of atrial tissue to a less diseased, fibrotic state or through other effects (*i.e.* on autonomic inputs to atrial tissue) is speculative. What is clear, however, is that a variety of oral agents have been linked to reduced AF burdens in diabetic patients [133]. The use of metformin and thiazolidinediones have been shown to reduce the incidence of new-onset AF in large cohort studies that may be related to decreased oxidative stress and atrial fibrosis [134, 135]. Finally, newer diabetes medication classes including glucagon-like peptide-1 receptor agonists (GLP-1RA), dipeptidyl peptidase-4 (DPP-4) inhibitors, and sodium-glucose cotransporter-2 (SGLT-2) inhibitors that have been associated with cardioprotective effects have not been shown to reduce the incidence of AF [133]. Given the decreased risk of major adverse cardiovascular events as seen with the use of GLP-1RA and SGLT-2 inhibitors, additional studies are urgently needed for newer classes of diabetes medications to assess the incidence of AF through potential interactions between oxidative stress, atrial fibrosis, and autonomic remodeling.

## CONCLUSION

AF typically progresses from a predominantly trigger-driven arrhythmia in which PV ectopy induces discrete episodes of PAF to a substrate-maintained arrhythmia in which ectopic atrial activity results in persistent fibrillatory activity stemming from slow conduction velocities and reduced atrial refractoriness. Because AF itself can increase arrhythmia susceptibility, interrupting the progression from PAF to persistent AF is critical if a rhythm-control strategy is to be pursued. Just as importantly, clinical risk factors including obesity, hypertension, OSA, and alcohol use contribute directly to electro-anatomical remodeling and trigger activity, increasing AF likelihood. Clinicians engaging in a rhythm-control strategy for AF patients not only need to target AF directly through antiarrhythmic medicines or catheter ablation but must target the clinical risk factors that contribute to AF risk.

## LIST OF ABBREVIATIONS

AF: Atrial Fibrillation
PAF or persAF: Paroxysmal or Persistent AF
PVI: Pulmonary Vein Isolation
AERPs: Atrial Effective Refractory Periods
